# Beige fat is dispensable for the metabolic benefits associated with myostatin deletion

**DOI:** 10.1016/j.molmet.2020.101120

**Published:** 2020-11-18

**Authors:** François Marchildon, Jingyi Chi, Sean O'Connor, Hilary Bediako, Paul Cohen

**Affiliations:** Laboratory of Molecular Metabolism, The Rockefeller University, 1230 York Avenue, New York City, NY 10065, USA

**Keywords:** Muscle hypertrophy, Beige adipocytes, Obesity, Insulin resistance, Hepatic steatosis, Metabolic dysfunction

## Abstract

**Objective:**

Increasing muscle mass and activating beige fat both have great potential for ameliorating obesity and its comorbidities. Myostatin null mice have increased skeletal muscle mass and are protected from obesity and its sequelae. Deletion of myostatin has also been suggested to result in the activation of beige adipocytes, thermogenic fat cells with anti-obesity and anti-diabetes properties. It is not known whether beige fat activation contributes to the protection from obesity in myostatin null mice.

**Methods:**

To investigate the role of beige fat activation in the metabolic benefits associated with myostatin deletion, we crossed myostatin null mice to adipocyte-specific PRDM16 knockout mice. We analyzed this new mouse model using molecular profiling, whole mount three-dimensional tissue imaging, tissue respiration, and glucose and insulin tolerance tests in models of diet-induced obesity.

**Results:**

Here, we report that PRDM16 is required for the activation of beige fat in the absence of myostatin. However, we show in both male and female mice that beige fat activation is dispensable for the protection from obesity, glucose intolerance, insulin resistance, and hepatic steatosis mediated by myostatin deletion.

**Conclusion:**

These findings demonstrate that increasing muscle mass can compensate for the inactivation of beige fat and raise the possibility of targeting muscle mass as a therapeutic approach to offset the deleterious effects of adipose tissue dysfunction in obesity and metabolic syndrome.

## Highlights

• Expression of PRDM16 is required for beige fat activation in myostatin null mice.
• Myostatin null mice remain protected from obesity and insulin resistance even in the absence of beige fat.
• Beige fat is not required for the protection from hepatic steatosis in myostatin null mice.

## 1 Introduction

Obesity currently affects approximately 650 million adults worldwide and is a major risk factor for type 2 diabetes, hypertension, dyslipidemia, coronary heart disease, and many types of cancer [1]. Obesity is also associated with diminished quality of life and increased risk for early death [2]. Of particular concern, the prevalence of overweight and obesity continues to increase, with more than 1 billion adults worldwide predicted to be obese by 2030 [3].

There are currently no highly effective medical therapies for obesity. Excess adiposity develops as a result of a chronic imbalance between energy intake and energy expenditure, and thus durable weight loss requires decreased food intake and/or increased energy utilization. Skeletal muscle constitutes 40% of body mass, and at rest, accounts for 30% of whole-body energy expenditure [4]. Upon insulin stimulation, 80% of glucose disposal occurs in skeletal muscle, and muscle mass is correlated with improved insulin sensitivity and protection from type II diabetes and metabolic syndrome in humans [5]. As a possible therapeutic approach, maintaining muscle mass by exercise training is well established to improve glycemic control, insulin sensitivity, and metabolic syndrome [6]. However, aging is associated with both increased adiposity and muscle wasting, and sarcopenic obesity is a major contributor to a constellation of chronic diseases [7].

Myostatin is a circulating protein that negatively regulates skeletal muscle mass by binding and activating the activin A receptor type IIB (ActRIIB) on the sarcolemma. Myostatin null mice show remarkable skeletal muscle hypertrophy, as do dogs, cows, and humans with mutations in this gene [8]. In clinical trials, agents that antagonize myostatin function have been reported to increase lean mass in muscular dystrophy patients [9]. Myostatin mutant (Mstn^-/-^) mice are also protected from obesity and its sequelae [[10], [11], [12]]. Despite this dramatic anti-obesity phenotype, the underlying molecular basis by which myostatin deletion protects from obesity remains unknown.

Mstn^-/-^ mice have more recently been reported to exhibit increased browning of the white fat [13,14], a phenotype generally resulting from increased brown-like fat cells that are present within subcutaneous white adipose tissues. These thermogenic beige adipocytes express uncoupling protein 1 (UCP1) and dissipate chemical energy as heat. The function of these cells is dependent on the transcriptional coregulatory protein PRDM16 [15]. Mice with an adipocyte-specific knockout of PRDM16 have an ablation of beige fat function and develop obesity, insulin resistance, hepatic steatosis, and increased adipose tissue inflammation. Conversely, mice with transgenic overexpression of PRDM16 in adipose tissue have increased beige fat activation and are protected from obesity and metabolic dysfunction [16]. Importantly, humans have been shown to possess inducible brown fat, that shows strong molecular similarities to rodent beige fat [[17], [18], [19], [20]]. The activation of these tissues by cold exposure and beta-3 adrenergic agents in humans is associated with anti-obesity and anti-diabetes effects [21,22].

The increased beige fat noted in Mstn^-/-^ mice raises the possibility that this tissue is responsible for the protection from obesity and its complications in the absence of myostatin [23]. We tested this hypothesis by crossing myostatin null mice (Mstn^-/-^) with adipocyte-specific PRDM16 knockout mice (Adipo-PRDM16 KO). We report that the expression of PRDM16 is an absolute requirement for the activation of beige adipocytes in the absence of myostatin. Using models of diet-induced obesity in male and female mice, however, we report that double knockout (dKO) mice remain protected from obesity and its sequelae irrespective of beige adipocytes.

## 2 Materials and methods

### 2.1 Animal studies and mouse models

All animal studies were performed in accordance with the institutional guidelines of The Rockefeller University Institutional Animal Care and Use Committee (IACUC) (Protocol #18016-H). All mice were provided food and water *ad libitum* with standard rodent diet (Labdiet Picolab Rodent 5053) or with a high-fat rodent diet (Research Diets Opensource D12492) as indicated. All mouse studies were performed in an animal facility maintained at 22 °C and 50% humidity level, with a 12-h light cycle (lights on at 07h00 and off at 19h00). Mice were housed 3 to 5 animals per cage. Average weekly body weight measurements are presented. For experiments in cold and thermoneutral conditions, cages were transferred to 8 °C or 30 °C incubators. Mice were housed in pairs at 8 °C. On the day longitudinal experiments ended, body composition was measured using an Echo MRI (Echo-MRI 100H). Adiposity and leanness were calculated by dividing fat or lean mass by body weight. Tissue wet weights were measured at necropsy using a standard laboratory scale. The myostatin null mouse model was provided by Dr. Se-Jin Lee [8]. The Adipo-PRDM16 KO mouse model has been previously reported [15]. All mouse lines were maintained on a C57BL/6 genetic background.

### 2.2 Histology

Animals were euthanized by isoflurane overdose. Tissues were dissected, transferred into histology cassettes, and fixed in 10% phosphate-buffered formalin for 36 h. Specimens were washed twice in phosphate-buffered saline (PBS) and stored in 70% ethanol. Tissues were processed by the Tri-Institutional Laboratory of Comparative Pathology. Briefly, specimens were dehydrated by alcohol gradient and xylene and embedded in paraffin. Sections were stained with hematoxylin and eosin using standard methods. Tissue sections were magnified with an upright brightfield microscope (Zeiss Axioplan 2), and micrographs were captured with a CMOS camera (Spot Imaging Insight CMOS) using standard microscopy techniques.

### 2.3 RNA purification, cDNA synthesis, and quantitative polymerase chain reaction (PCR)

Approximately 100 mg of WAT was collected at necropsy and flash frozen in liquid N_2_ and stored at −80 °C until processing. Specimens were resuspended in Trizol reagent (Invitrogen 15596026), homogenized mechanically, and RNA was separated with chloroform and purified on column (Qiagen RNeasy mini kit 74104). RNA quality and quantity were assessed by a spectrophotometer. Complementary DNA was synthesized by reverse transcriptase (Applied Biosystems High-Capacity cDNA Reverse Transcription Kit 4368814) from 2 μg of RNA. Quantitative PCR was done with SYBR green (Applied Biosystems Power SYBR™ Green PCR Master Mix 4368577) on a real-time thermocycler (ThermoFisher Scientific QuantStudio 6 Flex Real-Time PCR System) using a 384-well format. Gene expression was normalized to TATA-box binding protein and quantified using the 2^−ΔΔCT^ method.

### 2.4 Whole-tissue clearing and immunolabeling

The Adipo-Clear method has been reported previously [24]. Briefly, animals were euthanized by isoflurane overdose and cardiac perfused with PBS and 10% formalin, and tissues were dissected and fixed in 10% formalin for 18 h at 4 °C. Specimens were dehydrated in methanol gradients diluted into B1N buffer (0.1% Triton X100, 0.3 M glycine, 0.01% NaN_3_, pH7.4), delipidated with dichloromethane 3 times for 30 min each, washed in methanol, and rehydrated by the same methanol gradient as above. Specimens were then blocked in PTxwH (0.1% Triton X100, 0.05% Tween 20, 2 ug/ml Heparin, 0.01% NaN_3_) for 1 h and incubated with primary antibodies UCP1 (1:200, Abcam ab10983) and TH (1:200, Millipore AB1542) for 4 d. Specimens were washed 8 times for 5 min, 10 min, 15 min, 20 min, 1 h, 2 h, 4 h and 18 h in PTxwH and then incubated with secondary antibodies (Alexa568 anti-rabbit and Alexa647 anti-sheep, Invitrogen) for 4 d. Tissues were washed as above and then washed 3 times in PBS and embedded in agarose. Embedded specimens were dehydrated in a methanol gradient diluted in PBS, delipidated in dichloromethane 3 times for 1 h each and cleared in dibenzyl ether for 1 h. For imaging, we used a light-sheet microscope (Ultramicroscope II LaVision Biotec) equipped with a sCMOs camera (Andor Neo). Images were acquired with InspectorPro software (LaVision), and whole tissue renderings were done with Imaris software.

### 2.5 Oxygen consumption with Clark-type electrode

Tissue oxygen consumption was measured with a Clark-type oxygen electrode. Following euthanasia, tissues were dissected and minced. Tissue homogenates were weighed, and quadruplicates were transferred in oxygenated PBS. Tissues were transferred and stirred into a respirometer microcell connected to an oxygen meter (Strathkelvin Instruments MS200A). A platinum cathode silver anode electrode connected by a saturated potassium chloride solution (Strathkelvin Instruments 1302) was used for oxygen recording, and the electrode was shielded with a polypropylene membrane jacket (Strathkelvin Instruments SI020). Thirty seconds of each recording was used to calculate the rate of oxygen consumption. Oxygen consumption rate was thereafter normalized to tissue sample weight.

### 2.6 Glucose and insulin tolerance tests

On the morning of the test, mice were weighed, individually caged with fresh hypocaloric wood chip bedding and food was removed. Six hours later, fasting blood glucose was measured by tail vein draw with a glucose meter (Nova Max Plus, Nova Diabetes Cares) and a glucose bolus (20% d-glucose) was injected intraperitoneally at 2 g/kg for the glucose tolerance test. Blood glucose was measured at the times indicated. For the insulin tolerance test, mice were weighed, caged and fasted as above. Fasting blood glucose was measured as above, and insulin (0.1 U/ml Novolin R) diluted in saline was injected intraperitoneally at 0.75 U/kg. Blood glucose was measured at the times indicated.

### 2.7 Liver triglyceride quantification

Liver triglycerides were purified by saponification alkaline hydrolysis and measured indirectly by glycerol quantification. Briefly, 0.1 g of flash frozen liver was homogenized with ethanolic potassium hydroxide (100% ethanol, 30% KOH at 2:1 ratio) and incubated for 18 h at 55 °C. Upon homogenization, 50% ethanol was added to bring the volume to 1 mL and vortexed. Following centrifugation, 700 uL of the supernatant was mixed with 500 μL of 50% ethanol and vortexed. To 200 μL of this solution, 215 uL of 1 M MgCl_2_ was added. Following centrifugation, glycerol content was assayed using a free glycerol kit (Sigma F6428), and values were determined using a glycerol standard curve (Sigma G7793).

### 2.8 Plasma measurements

Following euthanasia by isoflurane overdose, blood was drawn by cardiac puncture and immediately transferred into a lithium heparin plasma tube. Plasma was separated from erythrocytes by centrifugation and flash frozen in liquid N_2_ and stored until analysis. Myostatin (R&D systems DGDF80) and insulin (Crystal Chem 90080) levels were determined by enzyme-linked immunosorbent assay (ELISA). Triglyceride (Cayman Chemical 10010303), non-esterified fatty acid (Fujifilm WAKO Diagnostics 999–34691) and lactate (Sigma MAK064) levels were determined by coupled enzymatic assays.

### 2.9 Statistics

For each animal experiment, the exact number of animals studied is indicated in the figure legend. For mRNA expression and oxygen consumption rate figures, each point represents one animal, the bar represents the group mean and the error bar represents the standard error of the mean (SEM). For body weights, glucose tolerance test (GTT) and insulin tolerance test (ITT) figures, each point represents the group mean, and the error bar represents the SEM. Where no error bar is found, the SEM magnitude is smaller than the point width. For histological measurement of adipocytes, 3 animals per genotype were included, and no fewer than 304 adipocytes for chow-fed animals or 107 adipocytes for high-fat diet (HFD)-fed animals were measured. For endpoint measurements, either an unpaired student t-test or one-way analysis of variance (ANOVA) test assuming a Gaussian distribution and equal dispersion were performed, followed by *post hoc* (Mstn^-/-^ vs Adipo-PRDM16 KO; Mstn^-/-^) Tukey honest significance difference tests. For the experiments with 4 groups of mice, our statistical analyses focused on comparisons of Mstn^-/-^ vs Adipo-PRDM16 KO; Mstn^-/-^ mice, as this was our primary experimental question, but the absence of notation of statistical significance over other groups does not imply a lack of significance. For longitudinal measurements, two-way ANOVA tests assuming a Gaussian distribution and equal dispersion were performed, followed by *post hoc* Tukey honest significance difference tests.

## 3 Results

### 3.1 Prdm16 is required for beige fat activation in myostatin null mice

Myostatin null mice, in addition to remarkable skeletal muscle hypertrophy, are protected from obesity and metabolic dysfunction and demonstrate browning of the white fat [10,13]. To investigate the role of beige fat in the metabolic benefits associated with myostatin deletion, we characterized adipose tissues from male and female myostatin null mice. Histological examination of the subcutaneous inguinal white adipose tissue (iWAT) revealed that adipocytes were markedly smaller in myostatin null mice (Supplementary Figure 1A). RNA levels of thermogenic (*Ppargc1a, Ppargc1b, Dio2, Ucp1*), brown/beige adipocyte-enriched (*Cidea, Cpt1b, Elovl3*) and mitochondrial electron transport chain (ETC) (*Cox4i1*) genes were significantly upregulated in iWAT of myostatin null mice, whereas the expression of *Prdm16*, a key regulator of beige adipocyte identity, was similar between genotypes (Supplementary Figure 1B). Adipocyte size was also decreased in the visceral epididymal white adipose tissue (eWAT) in myostatin null mice relative to controls, with associated upregulation of RNAs encoding thermogenic, brown/beige adipocyte-enriched, and mitochondrial ETC genes (Supplementary Figure 1, C and D). We observed similar findings in female mice, in which subcutaneous mammary white adipose tissue (mWAT) and visceral parametrial white adipose tissue (pWAT) demonstrated smaller adipocytes and increased RNA levels of thermogenic, brown/beige-enriched, and mitochondrial ETC genes in myostatin null animals (Supplementary Figure 2, A-C). Taken together, these data are consistent with increased beige fat activation in the absence of myostatin. Interscapular brown adipose tissue (iBAT), on the other hand, showed no histological or molecular differences between the two genotypes in either sex (Supplementary Figure 1, E and F, and Supplementary Figure 2D).

**Figure 1:**
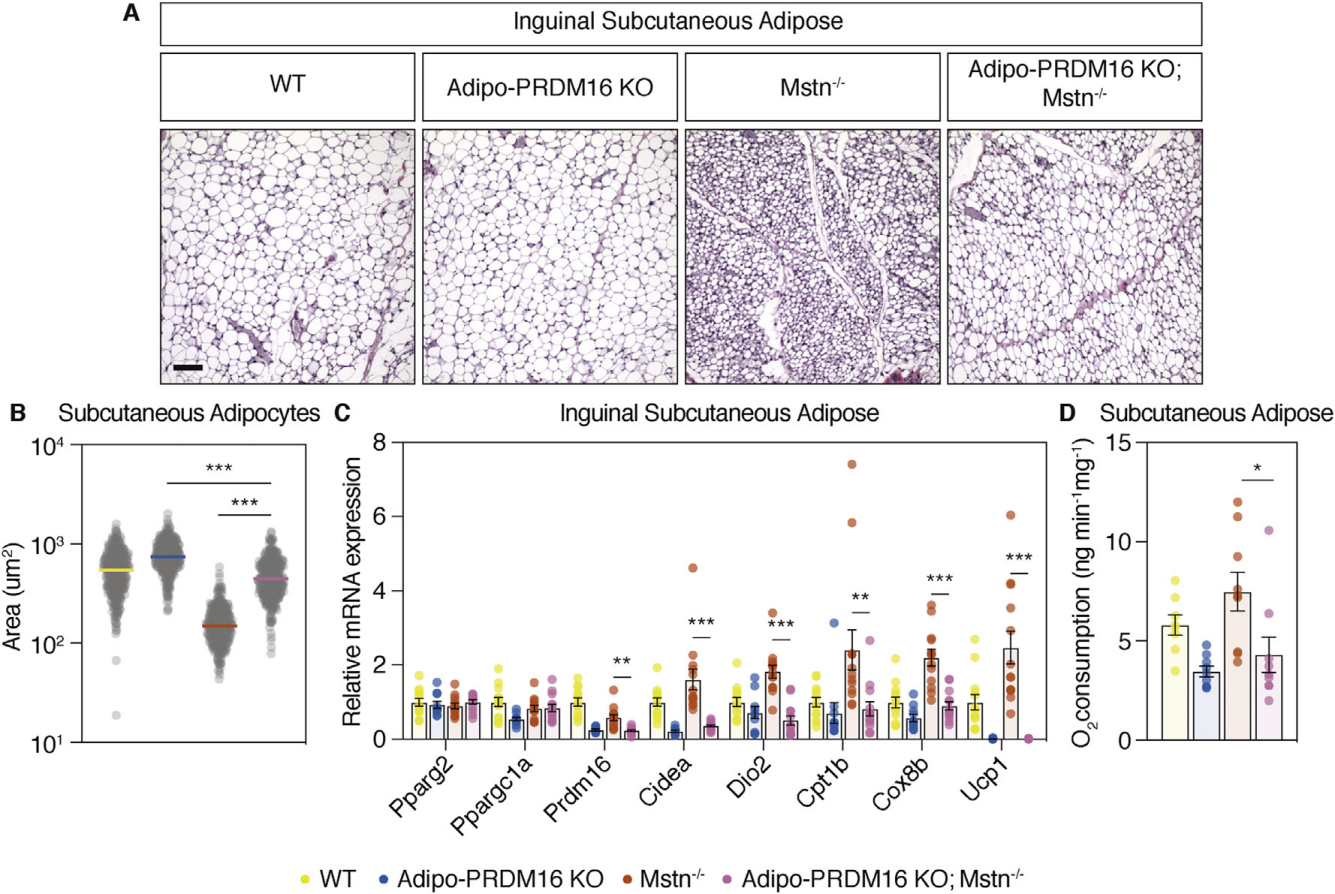
**Browning of the iWAT in myostatin null mice requires PRDM16 expression.** The iWAT of 10-week-old male Adipo-PRDM16 KO; Mstn^-/-^ double mutant and controls was studied. **(A)** Brightfield micrograph of iWAT stained with H&E (scale bar = 100um). n = 3 per group with representative images shown. **(B)** Each point represents one measurement of adipocyte area. The horizontal bars represent the median. n = 3, ∗∗∗p < 0.001 by one-way ANOVA with Tukey post-hoc test. **(C)** mRNA expression of thermogenic, brown/beige adipocyte-enriched, and mitochondrial electron transport chain genes determined by qPCR. n = 10–14 per group, ∗∗p < 0.01 ∗∗∗p < 0.001 by one-way ANOVA with Tukey post-hoc test. **(D)***Ex vivo* oxygen consumption measured with a Clark-type electrode. n = 8–9 per group. ∗p < 0.05 by one-way ANOVA with Tukey post-hoc test.

The transcriptional coregulatory protein PRDM16 is required for beige fat activation, which is associated with protection from obesity and improved insulin sensitivity [15,16]. To determine whether PRDM16 is required for increased beige fat biogenesis in the absence of myostatin, we crossed myostatin null (Mstn^-/-^) mice with adipocyte-specific PRDM16 knockout mice (Adipo-PRDM16 KO) to generate double mutants (Adipo-PRDM16 KO; Mstn^-/-^) [8,15]. The iWAT from Adipo-PRDM16 KO; Mstn^-/-^ mice was compared to Adipo-PRDM16 KO, Mstn^-/-^ and wild-type (WT) controls. Adipocytes were significantly larger in double mutant Adipo-PRDM16 KO; Mstn^-/-^ mice compared to Mstn^-/-^ (Figure 1A), with a 2.9-fold increase in area (Figure 1B). We then measured RNA levels of thermogenic, brown/beige adipocyte-enriched and ETC genes in these 4 groups of mice (Figure 1C). Compared to Mstn^-/-^ animals, the expression of *Cidea* (4.5-fold), *Dio2* (3.6-fold), *Cpt1b* (2.9-fold), *Cox8b* (2.4-fold), and *Ucp1* (159-fold) were significantly decreased in Adipo-PRDM16 KO; Mstn^-/-^ mice. Given this strong repression of *Ucp1* expression in the iWAT of Adipo-PRDM16 KO; Mstn^-/-^ mice, we analyzed UCP1 protein using whole mount Adipo-Clear imaging [24]. After housing at 8 °C for 48 h, Mstn^-/-^ tissues showed clusters of UCP1-positive cells, which were completely absent in Adipo-PRDM16 KO; Mstn^-/-^ mice (Supplementary Figure 3A and Movie S1). To assess whether these molecular changes result in functional differences, we analyzed *ex vivo* tissue respiration and demonstrated a 1.7-fold decrease in oxygen consumption in iWAT from Adipo-PRDM16 KO; Mstn^-/-^ mice relative to Mstn^-/-^ (Figure 1D).

Supplementary video related to this article can be found at https://doi.org/10.1016/j.molmet.2020.101120

The following is the supplementary data related to this article:

Movie S1 **3D visualization of beige fat activation in the iWAT Adipo-PRDM16 KO; Mstn**^**-/-**^**.** WT, Adipo-PRDM16 KO, Mstn^-/-^ and Adipo-PRDM16 KO; Mstn^-/-^ male mice fed a chow diet and housed 48 h at 8 °C. Rotating 3D projections of the iWAT immunolabeled with UCP1 (green) and autofluorescence (magenta).

Visceral adipose tissue is strongly associated with the metabolic syndrome [25] and is particularly resistant to beige fat activation [26]. As we measured an induction of beige/brown, thermogenic and ETC gene expression in eWAT of myostatin null mice, we asked if PRDM16 expression was required for beige fat activation in that depot. RNA levels of many beige/brown, thermogenic, and ETC genes were decreased in Adipo-PRDM16 KO; Mstn^-/-^ mice relative to Mstn^-/-^ (Supplementary Figure 4A). Furthermore, oxygen consumption was significantly decreased (1.9-fold) in eWAT of Adipo-PRDM16 KO; Mstn^-/-^ mice relative to Mstn^-/-^ (Supplementary Figure 4B). However, with Adipo-Clear imaging we were unable to detect any UCP1+ beige adipocytes in any of the genotypes, even after 7 d at 8 °C (Supplementary Figure 4C and Movie S2).

Supplementary video related to this article can be found at https://doi.org/10.1016/j.molmet.2020.101120

The following is the supplementary data related to this article:

Movie S2 **3D visualization of beige fat activation in the eWAT Adipo-PRDM16 KO; Mstn**^**-/-**^**.** WT, Adipo-PRDM16 KO, Mstn^-/-^ and Adipo-PRDM16 KO; Mstn^-/-^ male mice fed a chow diet and housed 7 days at 8 °C. Rotating 3D projections of the eWAT immunolabeled with UCP1 (green) and autofluorescence (magenta).

The iBAT is largely unaffected in Adipo-PRDM16 KO mice, presumably because *Prdm16* deletion is driven by adiponectin-Cre, which is expressed in late embryogenesis and postnatally, after iBAT has already developed [15]. Moreover, deletion of PRDM16 in developing iBAT, driven by Myf5-Cre, results in a very modest phenotype because of compensation by PRDM3. Myf5-Cre mediated deletion of both PRDM16 and PRDM3 results in a fairly dramatic iBAT phenotype [27]. Brown adipocyte activation has also been reported in the setting of myostatin deletion [23,28]. We therefore studied the iBAT in Adipo-PRDM16 KO; Mstn^-/-^ mice to determine whether loss of PRDM16 expression affects brown adipocyte morphology or function. iBAT histology showed more prominent lipid droplets in Adipo-PRDM16 KO mice, but no clear differences across the other 3 genotypes (Supplementary Figure 5A). We then measured the iBAT RNA levels of thermogenic, brown/beige adipocyte-enriched, and ETC genes in these 4 groups of mice (Supplementary Figure 5B). Only the expression of *Cox8b* (2.3-fold) was significantly decreased in Adipo-PRDM16 KO; Mstn^-/-^ mice compared to Mstn^-/-^.

### 3.2 Myostatin null mice remain protected from obesity and insulin resistance in the absence of beige fat

**Figure 2:**
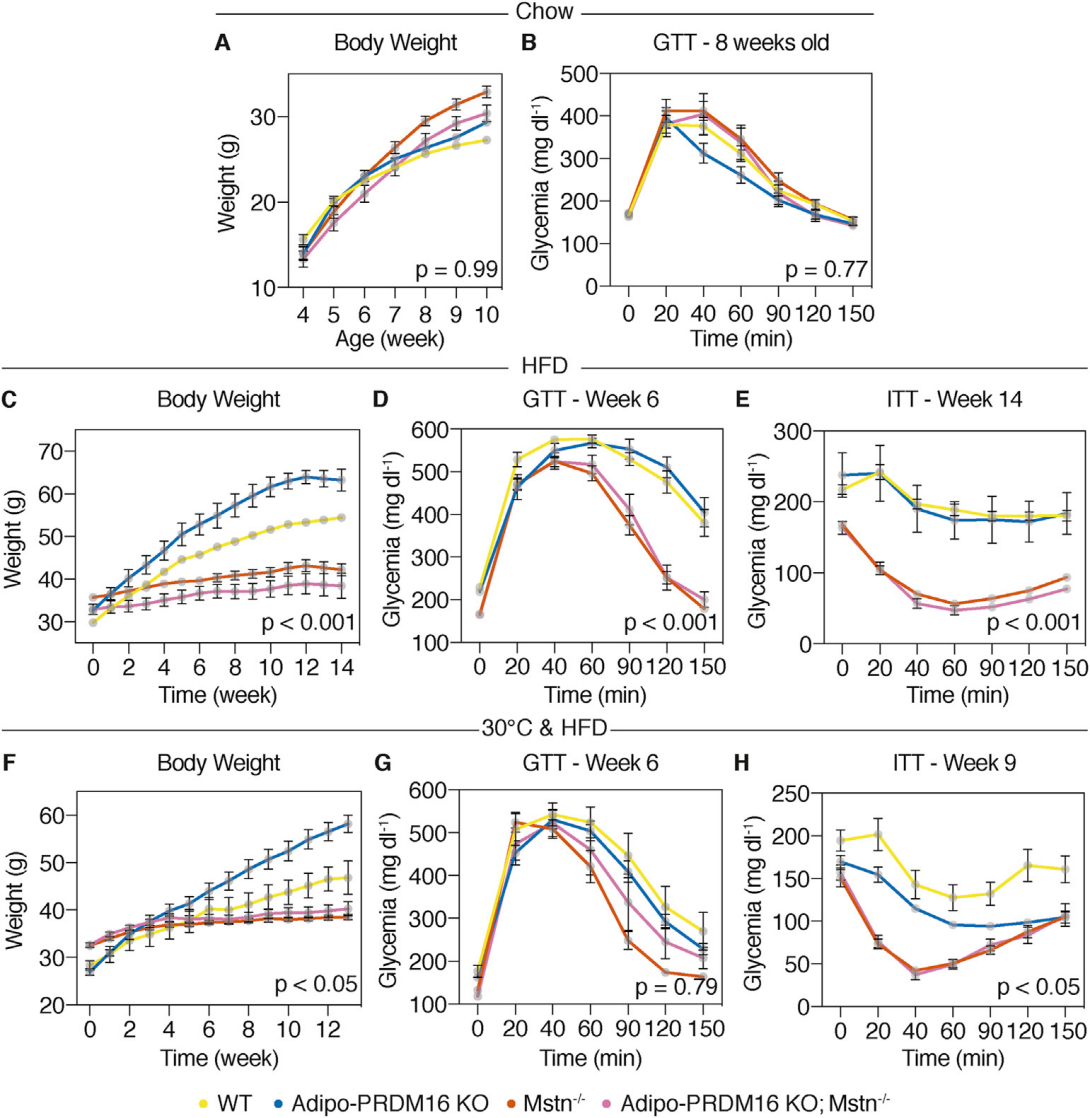
**Myostatin-null mice are protected from weight gain, hyperglycemia, and insulin resistance independent of beige adipocytes.** Male Adipo-PRDM16 KO; Mstn^-/-^ and control mice were studied starting at 4 weeks of age. At 10 weeks of age, mice were feed a HFD. **(A)** Body weight of mice on chow diet. **(B)** Intraperitoneal GTT [2 g/kg] on 8-week-old mice. **(C)** Body weight of mice on HFD. **(D)** Intraperitoneal GTT [2 g/kg] at 6 weeks on HFD. **(E)** ITT [0.75 U/kg] at 14 weeks on HFD. For A-E, n = 7–15 per group. **(F)** Another cohort of male Adipo-PRDM16 KO; Mstn^-/-^ double mutant and controls were housed at 30 °C at 6 weeks of age. At 10 weeks of age, mice were fed a HFD. Body weight of mice placed on HFD. **(G)** Intraperitoneal GTT at 6 weeks on HFD. **(H)** ITT at 9 weeks on HFD. For F–H, n = 5–11 per group. p-values indicate a two-way ANOVA with Tukey post-hoc test on Adipo-PRDM16 KO; Mstn^-/-^ vs. Adipo-PRDM16 KO.

Beige fat activation and skeletal muscle hypertrophy protect from obesity and its sequelae [10,16]. Having demonstrated the ablation of beige adipocytes in double mutant Adipo-PRDM16 KO; Mstn^-/-^ mice, we questioned whether the protection from obesity and its consequences in Mstn^-/-^ mice is dependent on beige fat activation. We first characterized a cohort of male Adipo-PRDM16 KO; Mstn^-/-^ and control mice on a standard chow diet (Table S1). Body weights were comparable between Adipo-PRDM16 KO; Mstn^-/-^ and Mstn^-/-^ mice, and these two groups showed no differences in fasting blood glucose, body composition or individual fat depot weights (Table S1). However, both Adipo-PRDM16 KO; Mstn^-/-^ and Mstn^-/-^ mice demonstrated significant differences in body composition relative to Adipo-PRDM16 KO mice. We then longitudinally measured body weight in a separate cohort of chow fed Adipo-PRDM16 KO; Mstn^-/-^ and control mice up to 10 weeks of age (Figure 2A). We assessed glucose homeostasis in Adipo-PRDM16 KO; Mstn^-/-^ and controls with an intraperitoneal GTT. Eight-week-old Adipo-PRDM16 KO; Mstn^-/-^ mice showed normal glycemia and comparable glucose tolerance to Mstn^-/-^ mice and the other groups (Figure 2B and Table S1).

We next characterized Mstn^-/-^ and Adipo-PRDM16 KO; Mstn^-/-^ mice on an obesogenic diet. Starting at 10 weeks of age, all 4 groups were fed a 60% HFD. Surprisingly, Adipo-PRDM16 KO; Mstn^-/-^ and Mstn^-/-^ were equally (Tukey post-hoc p = 0.15) and dramatically protected from obesity compared to Adipo-PRDM16 KO and WT control mice (Tukey post-hoc p < 0.001) (Figure 2C). Six weeks after beginning HFD, Adipo-PRDM16 KO; Mstn^-/-^ had comparable glucose tolerance compared to Mstn^-/-^ (Tukey post-hoc p = 0.95), which was significantly improved in both groups relative to Adipo-PRDM16 KO and WT controls (Tukey post-hoc p < 0.001) (Figure 2D). Fourteen weeks after starting HFD, we assessed insulin sensitivity by an intraperitoneal ITT. Adipo-PRDM16 KO; Mstn^-/-^ and Mstn^-/-^ showed comparable (Tukey post-hoc p = 0.92) but significantly improved insulin tolerance relative to Adipo-PRDM16 KO and WT controls groups (Tukey post-hoc p < 0.001) (Figure 2E).

To address the possibility that iBAT activation might confer partial protection from weight gain in Adipo-PRDM16 KO; Mstn^-/-^ mice, we raised Adipo-PRDM16 KO; Mstn^-/-^ and controls at thermoneutrality (30 °C) to suppress sympathetic output and non-shivering thermogenesis. At 10 weeks of age, all 4 groups of mice were fed HFD, and body weight was measured for 13 weeks. Adipo-PRDM16 KO; Mstn^-/-^ and Mstn^-/-^ mice showed similar body weight (Tukey post-hoc p = 0.96) and glucose (Tukey post-hoc p = 0.85) and insulin tolerance (Tukey post-hoc p = 0.99) at thermoneutrality (Figure 2F–H). Both Adipo-PRDM16 KO; Mstn^-/-^ and Mstn^-/-^ had significantly deceased body weight (Tukey post-hoc p < 0.05) and improved insulin sensitivity (Tukey post-hoc p < 0.05) compared to the other groups.

We also assessed the development of obesity and associated pathology in female Adipo-PRDM16 KO; Mstn^-/-^ mice. Body weight and composition were comparable between Adipo-PRDM16 KO; Mstn^-/-^ and Mstn^-/-^ female mice; however, we noted significant differences in body weight, adiposity and muscle mass between both groups and Adipo-PRDM16 KO control mice (Table S2). In another cohort of female Adipo-PRDM16 KO; Mstn^-/-^ and control mice, we measured a very small but statistically significant increase (1.1-fold) in body weight for Adipo-PRDM16 KO; Mstn^-/-^ compared to Mstn^-/-^ (Supplementary Figure 6A, Tukey post-hoc p < 0.001). Fasting glycemia and glucose tolerance were comparable between every group of female mice (Supplementary Figure 6B and Table S2). At 10 weeks of age, we fed female Adipo-PRDM16 KO; Mstn^-/-^ and control mice 60% HFD. As in males, female Adipo-PRDM16 KO; Mstn^-/-^ and Mstn^-/-^ had comparable body weight (Tukey post-hoc p = 0.10) and were equally protected from obesity compared to Adipo-PRDM16 KO and WT controls (Tukey post-hoc p < 0.001) (Supplementary Figure 6C and Table S3). Glucose (Tukey post-hoc p = 0.74) and insulin (Tukey post-hoc p = 0.92) tolerance were also comparable between Adipo-PRDM16 KO; Mstn^-/-^ and Mstn^-/-^ groups (Supplementary Figure 6D and 5E). Insulin tolerance was significantly improved in Adipo-PRDM16 KO; Mstn^-/-^ and Mstn^-/-^ compared to Adipo-PRDM16 KO and WT controls (Tukey post-hoc p < 0.01) (Supplementary Figure 6E).

The iBAT weight was comparable between Adipo-PRDM16 KO; Mstn^-/-^ and Mstn^-/-^ mice (Table 1). Histological examination of the iBAT showed comparable lipid accumulation in Adipo-PRDM16 KO; Mstn^-/-^ and Mstn^-/-^ mice, while both of these groups had significantly decreased lipid accumulation relative to WT and Adipo-PRDM16 KO controls (Supplementary Figure S5C). RNA levels of thermogenic, brown/beige adipocyte-enriched and ETC genes were comparable between these 4 groups, with only the expression of *Cox8b* (1.4-fold) significantly decreased in Adipo-PRDM16 KO; Mstn^-/-^ mice compared to Mstn^-/-^ (Supplementary Figure S5D).

**Table 1:** Phenotypic characterization of male Adipo-PRDM16 KO; Mstn^-/-^ double mutant and controls on HFD.

	WT (n = 14)	Adipo-PRDM16 KO (n = 7)	Mstn^-/-^ (n = 15)	Adipo-PRDM16 KO; Mstn^-/-^ (n = 9)	P-Value (dKO vs Adipo-PRDM16 KO)	P-Value (dKO vs Mstn^-/-^)
Body Weight (g)	47.14 ± 3.41	55.28 ± 4.37	37.31 ± 5.13	34.59 ± 5.73	<0.0001	NS
Food Consumption (kcal/day)	12.34 ± 3.75	10.40 ± 2.23	12.22 ± 2.22	9.78 ± 2.58	NS	NS
Adiposity (%)	37.40 ± 4.79	44.56 ± 3.28	7.02 ± 6.67	8.27 ± 7.92	<0.0001	NS
Body Fat Mass (g)	17.68 ± 2.92	24.74 ± 3.58	2.88 ± 3.06	3.22 ± 3.61	<0.0001	NS
Leanness (%)	57.23 ± 3.08	50.81 ± 4.07	88.67 ± 6.59	89.73 ± 8.24	<0.0001	NS
Body Non-Fat Mass (g)	26.94 ± 1.90	27.95 ± 1.22	32.83 ± 2.92	30.69 ± 3.18	NS	NS
Brown Adipose (g)	0.10 ± 0.02	0.07 ± 0.05	0.03 ± 0.01	0.03 ± 0.01	0.006	NS
Inguinal Adipose (g)	1.69 ± 0.35	1.78 ± 0.25	0.33 ± 0.27	0.48 ± 0.52	<0.0001	NS
Epididymal Adipose (g)	0.71 ± 0.13	0.96 ± 0.25	0.32 ± 0.29	0.19 ± 0.20	<0.0001	NS
Tibialis Anterior Muscle (g)	0.07 ± 0.01	0.06 ± 0.01	0.10 ± 0.01	0.10 ± 0.02	<0.0001	NS
Liver (g)	1.86 ± 0.26	1.70 ± 0.23	0.91 ± 0.17	0.87 ± 0.22	<0.0001	NS

Values are mean ± SD.

dKO: Double knockout.

### 3.3 Repression of adipose inflammation and hepatic steatosis in the Adipo-PRDM16 KO with loss of myostatin expression

Body composition analysis by magnetic resonance imaging (MRI) showed that lean and fat mass were comparable between male Adipo-PRDM16 KO; Mstn^-/-^ and Mstn^-/-^ controls (Table 1). We then performed a comprehensive necropsy of these mice, and for every measurement, Adipo-PRDM16 KO; Mstn^-/-^ mice were similar to Mstn^-/-^ (Table 1). However, both Adipo-PRDM16 KO; Mstn^-/-^ and Mstn^-/-^ mice had significantly decreased total fat mass (7.7-fold and 8.6-fold decrease) and iWAT mass (3.8-fold and 5.3-fold decrease) compared to Adipo-PRDM16 KO mice.

**Figure 3:**
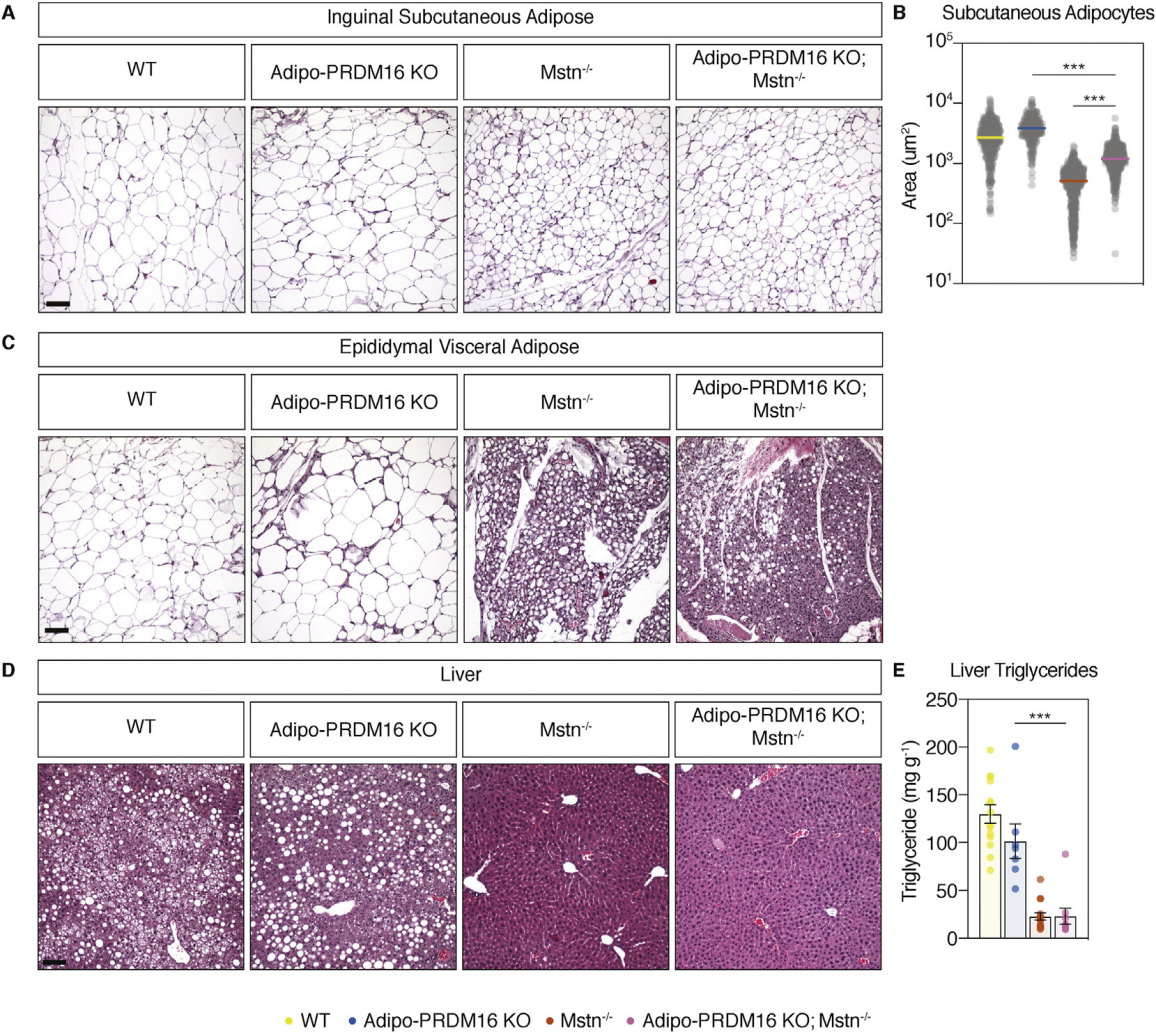
**Protection from adipocyte hypertrophy and hepatic steatosis in myostatin null mice is independent of beige fat activation.** Male Adipo-PRDM16 KO; Mstn^-/-^ and control mice fed 18 weeks on HFD. **(A)** Brightfield micrograph of iWAT stained with H&E (scale bar = 100 um). n = 3 per group with representative images shown. **(B)** iWAT adipocyte area. Each point represents one measurement. Horizontal bars represent the median. n = 3 per group, ∗∗∗p < 0.001 by one-way ANOVA with Tukey post-hoc test. **(C)** Brightfield micrograph of eWAT stained with H&E (scale bar = 100 um). n = 3 per group with representative images shown. **(D)** Brightfield micrograph of the liver stained by H&E (scale bar = 100 um). n = 3 per group with representative images shown. **(E)** Liver triglyceride quantification. n = 7–15 per group, ∗∗∗p < 0.001 by one-way ANOVA with Tukey post-hoc test.

After 18 weeks of HFD, the iWAT of Adipo-PRDM16 KO; Mstn^-/-^ mice showed markedly larger adipocytes compared to Mstn^-/-^ controls (Figure 3A), with a 2.5-fold increase in adipocyte area (Figure 3B). In visceral eWAT, both Mstn^-/-^ and Adipo-PRDM16 KO; Mstn^-/-^ mice had clearly decreased lipid accumulation compared to Adipo-PRDM16 KO and WT controls (Figure 3C). The decrease in lipid area in both Mstn^-/-^ and Adipo-PRDM16 KO; Mstn^-/-^ groups was associated with an increased cellular infiltrate and dense eosin staining.

Adipocyte hypertrophy is associated with adipose tissue inflammation. Since iWAT adipocytes of Adipo-PRDM16 KO; Mstn^-/-^ were significantly larger compared to Mstn^-/-^, we assessed inflammation in this depot by measuring RNA levels of monocyte/macrophage (*Cd11c*, *CD68*, *Ccl2*) and T cell (*Cd4*) markers as well as pro-inflammatory mediators (*Tnfa*, *Saa3*, *Agt*, *Rst*) (Supplementary Figure 7A). The expression of these inflammatory markers was similar between Adipo-PRDM16 KO; Mstn^-/-^ and Mstn^-/-^, with the exception of an upregulation of *Agt* (9.5-fold) in Adipo-PRDM16 KO; Mstn^-/-^ mice. On the other hand, the expression of *Cd11c* (9.9-fold), *CD68* (5.1-fold), *Ccl2* (4.8-fold), *Tnfa* (3.6-fold) and *Saa3* (9.9-fold) were significantly repressed in Adipo-PRDM16 KO; Mstn^-/-^ iWAT relative to Adipo-PRDM16 KO controls. In iBAT, gene expression of the monocyte/macrophage markers *Cd11c* (22.2-fold), *Cd68* (7.3-fold), *Ccl2* (12.3-fold) and the pro-inflammatory mediator *Tnfa* (6.1-fold) were significantly decreased in Adipo-PRDM16 KO; Mstn^-/-^ mice relative to Adipo-PRDM16 KO controls (Supplementary Figure S7B).

Obesity and insulin resistance promote the development of nonalcoholic fatty liver disease (NAFLD). At necropsy, we noted decreased liver weight (1.9-fold lighter) in Adipo-PRDM16 KO; Mstn^-/-^ mice compared to Adipo-PRDM16 KO controls (Table 1). Histological and biochemical examination showed that both Adipo-PRDM16 KO; Mstn^-/-^ and Mstn^-/-^ had significantly decreased hepatic triglyceride accumulation (Figure 3D,E).

We also analyzed blood from Adipo-PRDM16 KO; Mstn^-/-^ male mice and controls fed HFD for 18 weeks (Table S4). For every analyte, male Adipo-PRDM16 KO; Mstn^-/-^ were comparable to Mstn^-/-^ mice. However, Adipo-PRDM16 KO; Mstn^-/-^ and Mstn^-/-^ mice had significantly improved glucose and insulin levels compared to Adipo-PRDM16 KO controls, consistent with their improved glucose and insulin tolerance (Table S4). Fasting triglycerides, non-esterified fatty acids, and serum lactate were comparable across all groups (Table S4).

## 4 Discussion

Our studies here demonstrate that myostatin deletion results in the activation of beige fat, which we have confirmed by histology, three-dimensional tissue imaging, gene expression and tissue respiration. We then crossed adipocyte-specific PRDM16 knockout mice with an ablation of beige fat to myostatin null mice and found that PRDM16 is absolutely required for beige fat activation in the absence of myostatin. Although our results demonstrate that beige adipocytes are activated by loss of myostatin, how myostatin deletion results in beige fat activation remains unclear. Since myostatin is predominantly secreted by skeletal muscle, the effects of myostatin inactivation on beige fat are likely the result of inter-organ crosstalk. Treatment of obese mice with a decoy myostatin receptor has been shown to activate both brown and beige adipocytes [29,30]. Interestingly, in our study the expression of thermogenic genes in the interscapular brown adipose is similar between myostatin null mice and controls, suggesting that myostatin deletion may have distinct effects on brown and beige adipocyte activation, perhaps related to the different embryonic origin of these cell types [31].

Despite Adipo-PRDM16 KO; Mstn^-/-^ mice being incapable of activating beige fat, like Mstn^-/-^ animals, they remain remarkably protected from obesity, glucose intolerance and insulin resistance when fed an HFD. These data demonstrate that increasing muscle mass can compensate for the metabolic dysfunction associated with a loss of beige fat. It should be noted that despite Adipo-PRDM16 KO mice being incapable of activating thermogenic genes, Adipo-PRDM16 KO; Mstn^-/-^ mice have smaller adipocytes. Decreased adipocyte size in Adipo-PRDM16 KO; Mstn^-/-^ mice relative to Adipo-PRDM16 KO is likely indirect and might be the consequence of an increase in energy utilization by the muscle that is significantly more abundant in in myostatin null mice. Protection from obesity by increasing muscle mass is not unique to the myostatin pathway. For example, Akt1 overexpression in skeletal muscle can stimulate hypertrophy of glycolytic fibers, as is seen in myostatin-null muscle, and concomitant robust protection from obesity [32]. Newer clinical studies have also demonstrated that increased muscle mass is associated with improved insulin sensitivity, independent of fat mass [33].

Mstn^-/-^ and Adipo-PRDM16 KO; Mstn^-/-^ were also equally and significantly protected from hepatic steatosis, in accordance with prior studies examining the consequences of myostatin deletion in obesity models [34]. Our double mutant mouse model highlights the fact that hepatic steatosis seen earlier in the absence of beige fat can be rescued by the inactivation of myostatin. We speculate that protection from hepatic steatosis in Adipo-PRDM16 KO; Mstn^-/-^ mice might be due to increased glucose disposal and utilization by skeletal muscle. Indeed, like Mstn^-/-^ mice, Adipo-PRDM16 KO; Mstn^-/-^ show significantly improved glucose tolerance and insulin sensitivity on an HFD. The decrease in eWAT lipid content might also reflect an increase in glucose disposal in the skeletal muscle in Mstn^-/-^ and Adipo-PRDM16 KO; Mstn^-/-^ mice.

It is possible that PRDM16 has functions in adipocytes independent of its role as an activator of thermogenic genes and a repressor of white fat genes. The full spectrum of phenotypes regulated by PRDM16 and beige adipocytes is still not fully understood. Our work here demonstrates that increasing muscle mass is sufficient to offset many of the sequalae associated with loss of beige adipocytes.

## 5 Conclusion

Our data indicate that the remarkable protection from obesity and its sequelae seen in myostatin null mice is not dependent on the activation of beige fat. How myostatin deletion leads to these dramatic, systemic metabolic benefits remains an area of ongoing investigation. We postulate that these benefits are mediated by both increased energy expenditure and altered endocrine signals in the absence of myostatin. Our findings suggest that therapeutically inhibiting myostatin could have broad benefits in the large population of patients with obesity and metabolic syndrome. More broadly, our work supports a growing body of literature indicating that increasing skeletal muscle mass can offset the detrimental effects of obesity and adipose tissue dysfunction.

## Author contributions

F.M. and P.C. conceived the study. F.M., J.C., S.O. and H.B., performed experiments and analyzed data. F.M. and J.C. visualized the images. F.M. and P.C. wrote the manuscript, and all authors approved the final version. P.C. provided intellectual input and supervised the research.

## Appendix A Supplementary data

Multimedia component 1 **Loss of myostatin induces beige fat activation in male mice. Twelve-week-old Mstn**^**-/-**^**and WT control male mice were studied.****(A)** Brightfield micrograph of the iWAT stained with H&E (scale bar = 50 um). n = 6 per group, with representative images shown. **(B)** Relative mRNA expression of thermogenic, brown/beige adipocyte-enriched, and mitochondrial electron transport chain genes determined by qPCR. n = 8 per group. ∗p < 0.05, ∗∗p < 0.01 by unpaired t-test. **(C)** Brightfield micrograph of eWAT stained with H&E (scale bar = 50 um). n = 6 per group with representative images shown. **(D)** Relative mRNA expression of thermogenic, brown/beige adipocyte-enriched, and mitochondrial electron transport chain genes determined by qPCR. n = 8 per group. ∗p < 0.05, ∗∗p < 0.01 by unpaired t-test. **(E)** Brightfield micrograph of iBAT stained with H&E (scale bar = 50 um). n = 6 per group, with representative images shown. **(F)** Relative mRNA expression of thermogenic, brown/beige adipocyte-enriched, and mitochondrial electron transport chain determined by qPCR. n = 8 per group. Multimedia component 1

Multimedia component 2 **Loss of myostatin induces beige fat activation in female mice. Fourteen-week-old Mstn**^**-/-**^**and WT controls female mice were studied.****(A)** Brightfield micrograph of mWAT stained with H&E (scale bar = 50 um). n = 3 per group with representative images shown. **(B)** Relative mRNA expression of thermogenic, brown/beige adipocyte-enriched, and mitochondrial electron transport chain genes determined by qPCR. n = 6 per group. ∗p < 0.05, ∗∗p < 0.01, ∗∗∗p < 0.001 by unpaired t-test. **(C)** Brightfield micrograph of pWAT stained with H&E (scale bar = 50 um). n = 3 per group with representative images shown. **(D)** Brightfield micrograph of iBAT stained with H&E (scale bar = 50um) n = 3 per group with representative images shown. Multimedia component 2

Multimedia component 3 **Beige fat activation in the iWAT of myostatin null mice is dependent on PRDM16. WT, Adipo-PRDM16 KO, Mstn**^**-/-**^**and Adipo-PRDM16 KO;Mstn**^**-/-**^**male mice fed a chow diet and housed 48 h at 8°C.****(A)** Fluorescent three-dimensional projections of iWAT from mice. Single channel for UCP1 (top), autofluorescence (middle) and overlay (bottom) is shown. n = 3 per group with representative images shown. Multimedia component 3

Multimedia component 4 **Beige fat activation in the eWAT of myostatin null mice is dependent on PRDM16. Ten-week-old Adipo-PRDM16 KO; Mstn**^**-/-**^**and control male mice fed a chow diet.****(A)** eWAT relative mRNA expression of thermogenic, brown/beige adipocyte-enriched, and mitochondrial electron transport chain genes determined by qPCR. n = 10-14 per group. ∗p < 0.05, ∗∗p < 0.01, ∗∗∗p < 0.001 by one-way ANOVA with Tukey post-hoc test. **(B)***Ex vivo* oxygen consumption measured with a Clark-type electrode. n = 8-9 per group. ∗∗p < 0.01 by one-way ANOVA with Tukey post-hoc test. **(C)** Fluorescent three-dimensional projections of eWAT from mice housed for 7 days at 6°C. Whole tissues were cleared and immunolabeled with UCP1 (top) and autofluorescence (bottom). n = 2 per group with representative images shown. Multimedia component 4

Multimedia component 5 **Effects of myostatin deletion in iBAT.** Ten-week-old male Adipo-PRDM16 KO; Mstn^-/-^ and control mice **(A)** Brightfield micrograph of iBAT stained with H&E (scale bar = 100 um). n = 3 per group with representative images shown. **(B)** mRNA expression of thermogenic, brown/beige adipocyte-enriched, and mitochondrial electron transport chain genes determined by qPCR. n = 10-14 per group, ∗∗p < 0.01 ∗∗∗p < 0.001 by one-way ANOVA with Tukey post-hoc test. **(C)** Male Adipo-PRDM16 KO; Mstn^-/-^ and control mice fed 18 weeks on HFD. Brightfield micrograph of iBAT stained with H&E (scale bar = 100 um). n = 3 per group, with representative images shown. **(D)** mRNA expression of thermogenic, brown/beige adipocyte-enriched, and mitochondrial electron transport chain genes determined by qPCR. n = 7-15 per group, ∗p < 0.05 ∗∗∗p < 0.001 by one-way ANOVA with Tukey post-hoc test. Multimedia component 5

Multimedia component 6 **Female myostatin-null mice are protected from weight gain, hyperglycemia, and insulin resistance independent of beige fat activation.** Female Adipo-PRDM16 KO;Mstn^-/-^ and control mice were studied starting at 4 weeks of age. At 10 weeks of age, mice were fed an HFD. **(A)** Body weight of mice on chow diet. **(B)** Intraperitoneal GTT [2 g/kg] on 8-week-old mice. **(C)** Body weight of mice fed an HFD. **(D)** Intraperitoneal GTT [2 g/kg] at 6 weeks. **(E)** ITT [0.75 U/kg] at 14 weeks on HFD. For A-E, n = 7-12 per group, p-value indicates a two-way ANOVA with Tukey post-hoc test on Adipo-PRDM16 KO;Mstn^-/-^ vs Adipo-PRDM16 KO. Multimedia component 6

Multimedia component 7 **Myostatin deletion protects from adipose inflammation independent of beige fat activation.** Male Adipo-PRDM16 KO; Mstn^-/-^ and control mice fed 18 weeks on HFD. **(A)** iWAT mRNA expression of monocyte/macrophage, T cell and pro-inflammatory markers determined by qPCR. n = 7-15 per group, ∗∗p < 0.01 ∗∗∗p < 0.001 by one-way ANOVA with Tukey post-hoc test. **(B)** iBAT mRNA expression of monocyte/macrophage, T cell and pro-inflammatory markers determined by qPCR. n = 7-15 per group, ∗p < 0.05 ∗∗p < 0.01 ∗∗∗p < 0.001 by one-way ANOVA with Tukey post-hoc test. Multimedia component 7
