# Supplementary figures and images for: Beige fat is dispensable for the metabolic benefits associated with myostatin deletion
Source: Mol Metab. 2020 Nov 18;43:101120. doi: 10.1016/j.molmet.2020.101120 (PMC7736974; doi:10.1016/j.molmet.2020.101120)

FIGURE S1

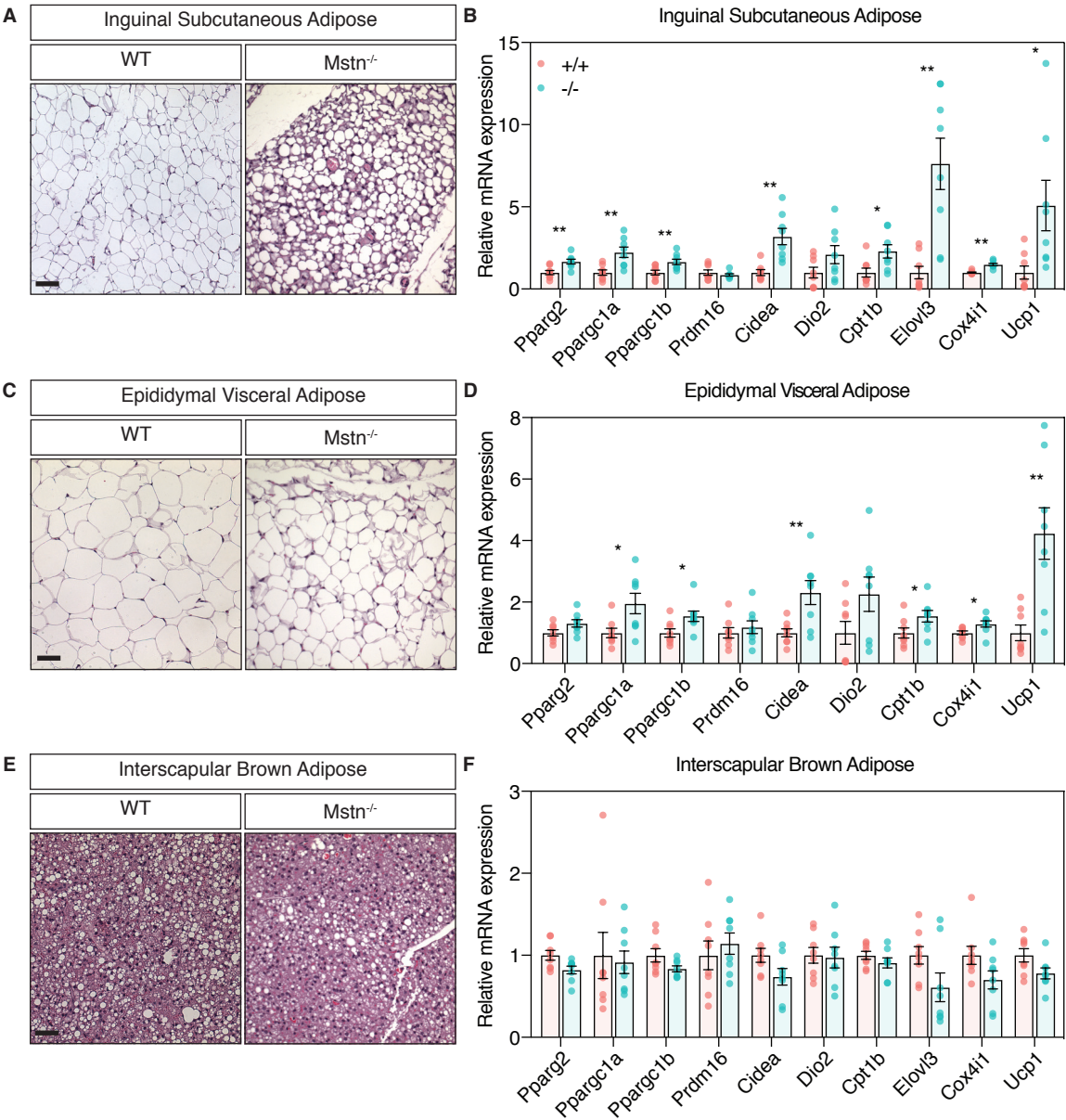

Supplement: Multimedia component 1 — Loss of myostatin induces beige fat activation in male mice. Twelve-week-old Mstn-/-and WT control male mice were studied.(A) Brightfield micrograph of the iWAT stained with H&E (scale bar = 50 um). n = 6 per group, with representative images shown. (B) Relative mRNA expression of thermogenic, brown/beige adipocyte-enriched, and mitochondrial electron transport chain genes determined by qPCR. n = 8 per group. ∗p < 0.05, ∗∗p < 0.01 by unpaired t-test. (C) Brightfield micrograph of eWAT stained with H&E (scale bar = 50 um). n = 6 per group with representative images shown. (D) Relative mRNA expression of thermogenic, brown/beige adipocyte-enriched, and mitochondrial electron transport chain genes determined by qPCR. n = 8 per group. ∗p < 0.05, ∗∗p < 0.01 by unpaired t-test. (E) Brightfield micrograph of iBAT stained with H&E (scale bar = 50 um). n = 6 per group, with representative images shown. (F) Relative mRNA expression of thermogenic, brown/beige adipocyte-enriched, and mitochondrial electron transport chain determined by qPCR. n = 8 per group. [file mmc1.pdf]

FIGURE S2

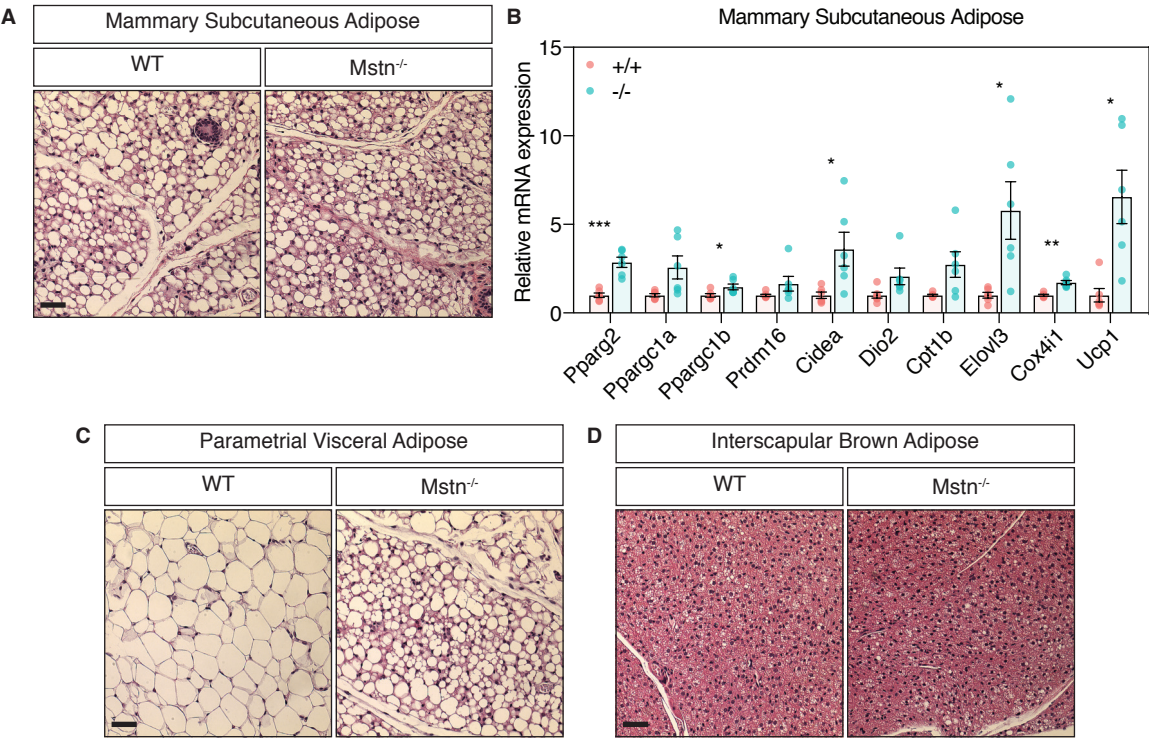

Supplement: Multimedia component 2 — Loss of myostatin induces beige fat activation in female mice. Fourteen-week-old Mstn-/-and WT controls female mice were studied.(A) Brightfield micrograph of mWAT stained with H&E (scale bar = 50 um). n = 3 per group with representative images shown. (B) Relative mRNA expression of thermogenic, brown/beige adipocyte-enriched, and mitochondrial electron transport chain genes determined by qPCR. n = 6 per group. ∗p < 0.05, ∗∗p < 0.01, ∗∗∗p < 0.001 by unpaired t-test. (C) Brightfield micrograph of pWAT stained with H&E (scale bar = 50 um). n = 3 per group with representative images shown. (D) Brightfield micrograph of iBAT stained with H&E (scale bar = 50um) n = 3 per group with representative images shown. [file mmc2.pdf]

FIGURE S3

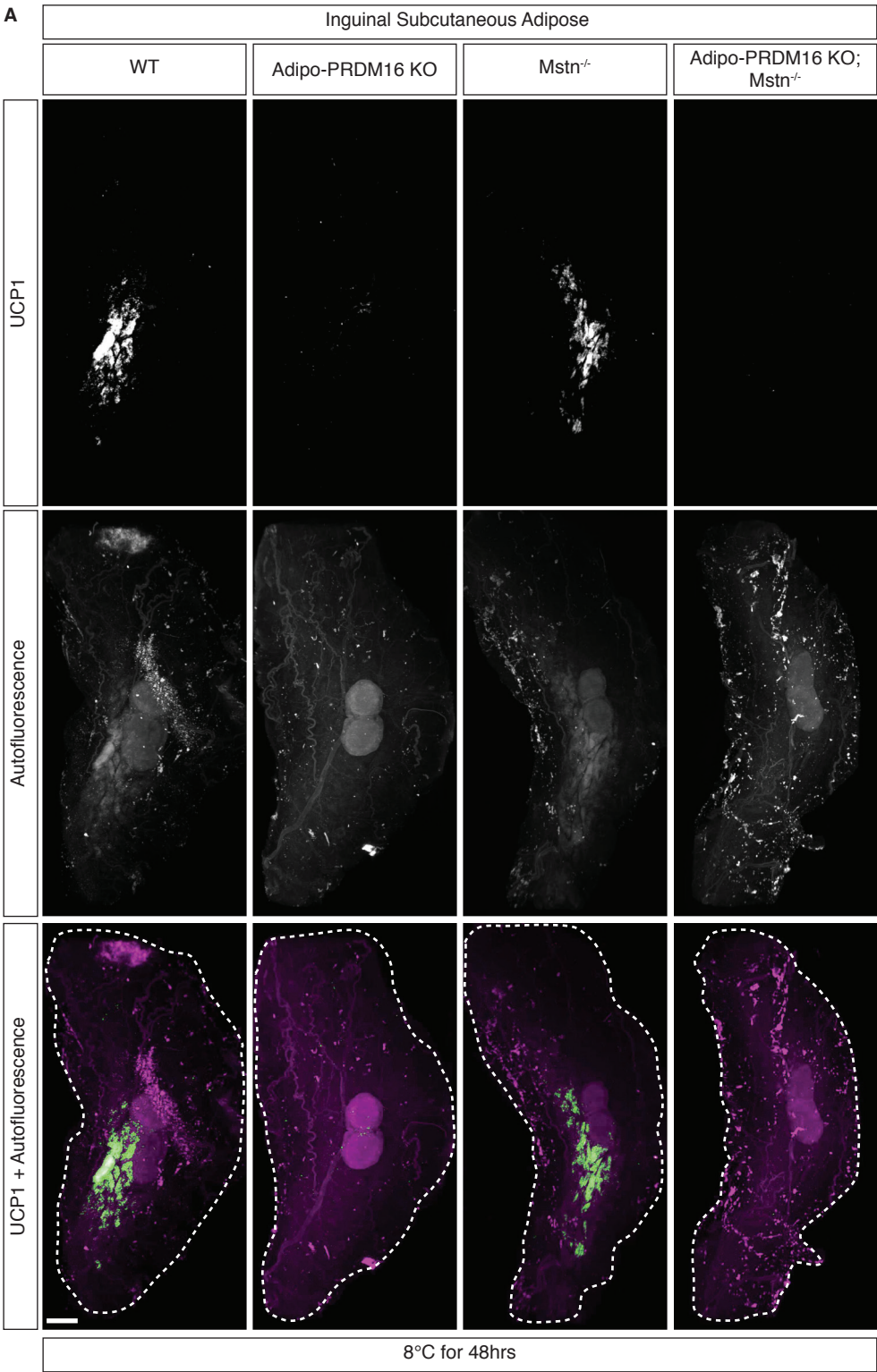

Supplement: Multimedia component 3 — Beige fat activation in the iWAT of myostatin null mice is dependent on PRDM16. WT, Adipo-PRDM16 KO, Mstn-/-and Adipo-PRDM16 KO;Mstn-/-male mice fed a chow diet and housed 48 h at 8°C.(A) Fluorescent three-dimensional projections of iWAT from mice. Single channel for UCP1 (top), autofluorescence (middle) and overlay (bottom) is shown. n = 3 per group with representative images shown. [file mmc3.pdf]

FIGURE S4

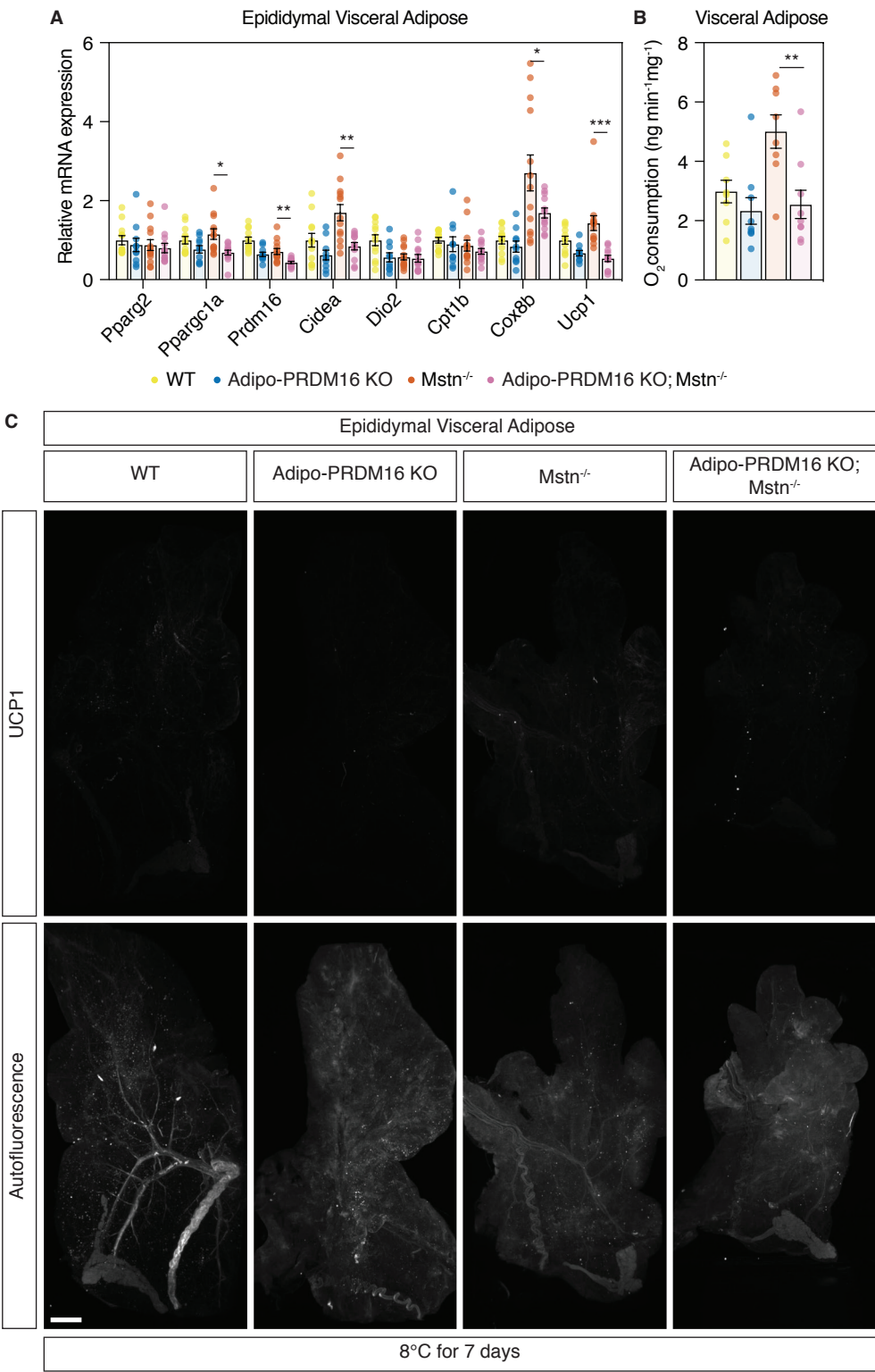

Supplement: Multimedia component 4 — Beige fat activation in the eWAT of myostatin null mice is dependent on PRDM16. Ten-week-old Adipo-PRDM16 KO; Mstn-/-and control male mice fed a chow diet.(A) eWAT relative mRNA expression of thermogenic, brown/beige adipocyte-enriched, and mitochondrial electron transport chain genes determined by qPCR. n = 10-14 per group. ∗p < 0.05, ∗∗p < 0.01, ∗∗∗p < 0.001 by one-way ANOVA with Tukey post-hoc test. (B)Ex vivo oxygen consumption measured with a Clark-type electrode. n = 8-9 per group. ∗∗p < 0.01 by one-way ANOVA with Tukey post-hoc test. (C) Fluorescent three-dimensional projections of eWAT from mice housed for 7 days at 6°C. Whole tissues were cleared and immunolabeled with UCP1 (top) and autofluorescence (bottom). n = 2 per group with representative images shown. [file mmc4.pdf]

FIGURE S5

Chow

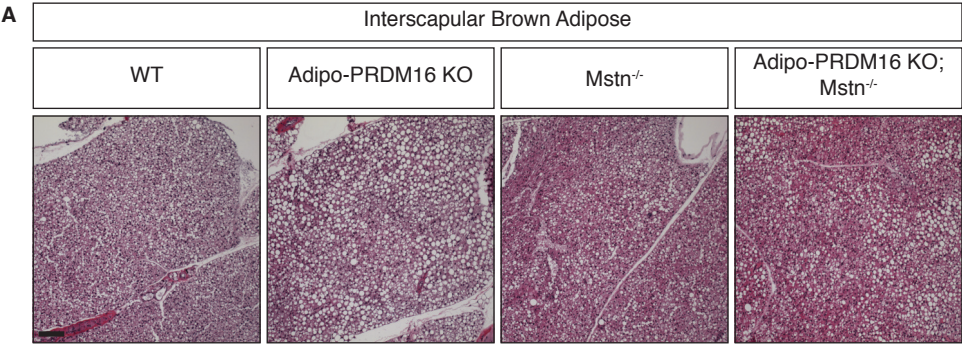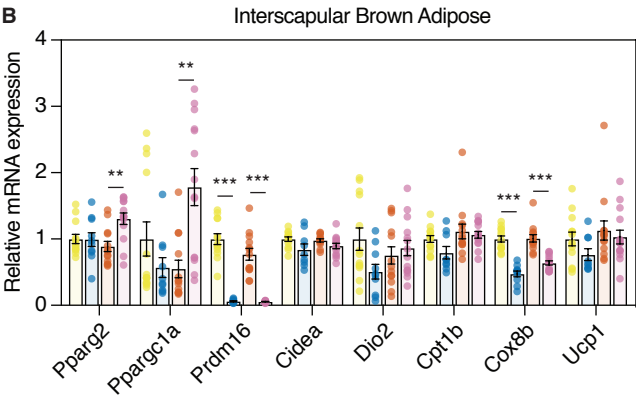

HFD

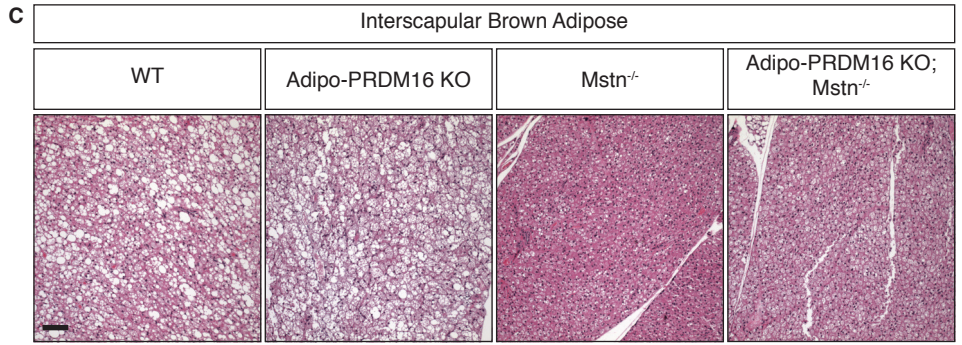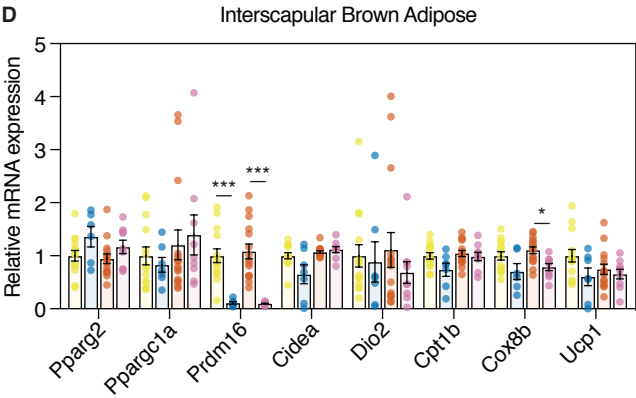

● WT ● Adipo-PRDM16 KO ● Mstn<sup>-/-</sup> ● Adipo-PRDM16 KO; Mstn<sup>-/-</sup>

Supplement: Multimedia component 5 — Effects of myostatin deletion in iBAT. Ten-week-old male Adipo-PRDM16 KO; Mstn-/- and control mice (A) Brightfield micrograph of iBAT stained with H&E (scale bar = 100 um). n = 3 per group with representative images shown. (B) mRNA expression of thermogenic, brown/beige adipocyte-enriched, and mitochondrial electron transport chain genes determined by qPCR. n = 10-14 per group, ∗∗p < 0.01 ∗∗∗p < 0.001 by one-way ANOVA with Tukey post-hoc test. (C) Male Adipo-PRDM16 KO; Mstn-/- and control mice fed 18 weeks on HFD. Brightfield micrograph of iBAT stained with H&E (scale bar = 100 um). n = 3 per group, with representative images shown. (D) mRNA expression of thermogenic, brown/beige adipocyte-enriched, and mitochondrial electron transport chain genes determined by qPCR. n = 7-15 per group, ∗p < 0.05 ∗∗∗p < 0.001 by one-way ANOVA with Tukey post-hoc test. [file mmc5.pdf]

FIGURE S6

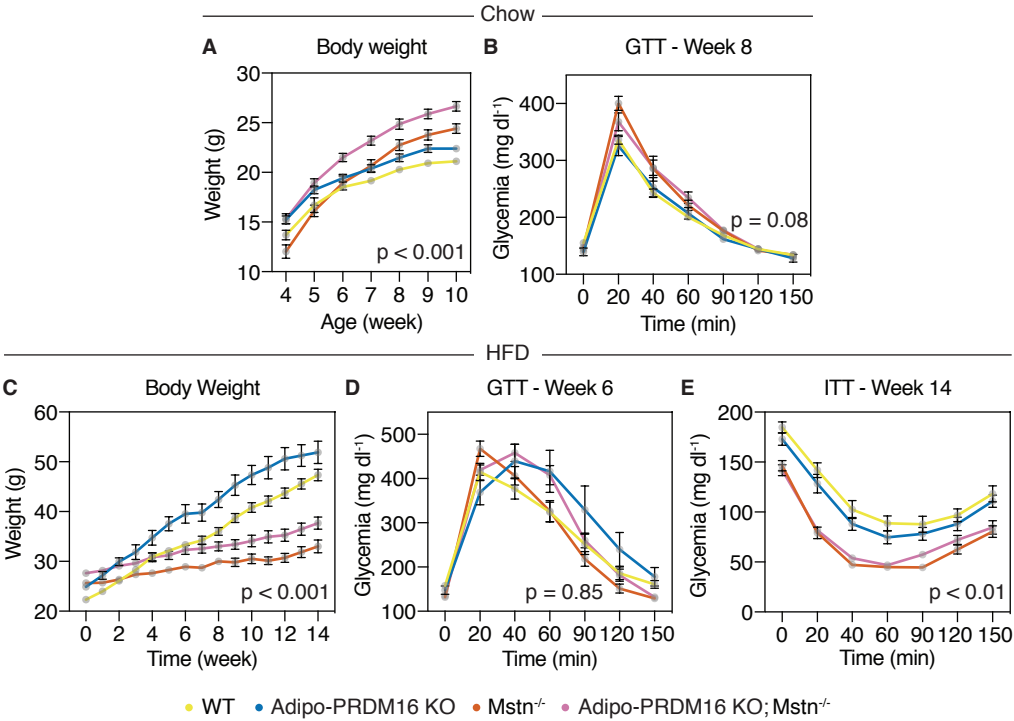

Supplement: Multimedia component 6 — Female myostatin-null mice are protected from weight gain, hyperglycemia, and insulin resistance independent of beige fat activation. Female Adipo-PRDM16 KO;Mstn-/- and control mice were studied starting at 4 weeks of age. At 10 weeks of age, mice were fed an HFD. (A) Body weight of mice on chow diet. (B) Intraperitoneal GTT [2 g/kg] on 8-week-old mice. (C) Body weight of mice fed an HFD. (D) Intraperitoneal GTT [2 g/kg] at 6 weeks. (E) ITT [0.75 U/kg] at 14 weeks on HFD. For A-E, n = 7-12 per group, p-value indicates a two-way ANOVA with Tukey post-hoc test on Adipo-PRDM16 KO;Mstn-/- vs Adipo-PRDM16 KO. [file mmc6.pdf]

FIGURE S7

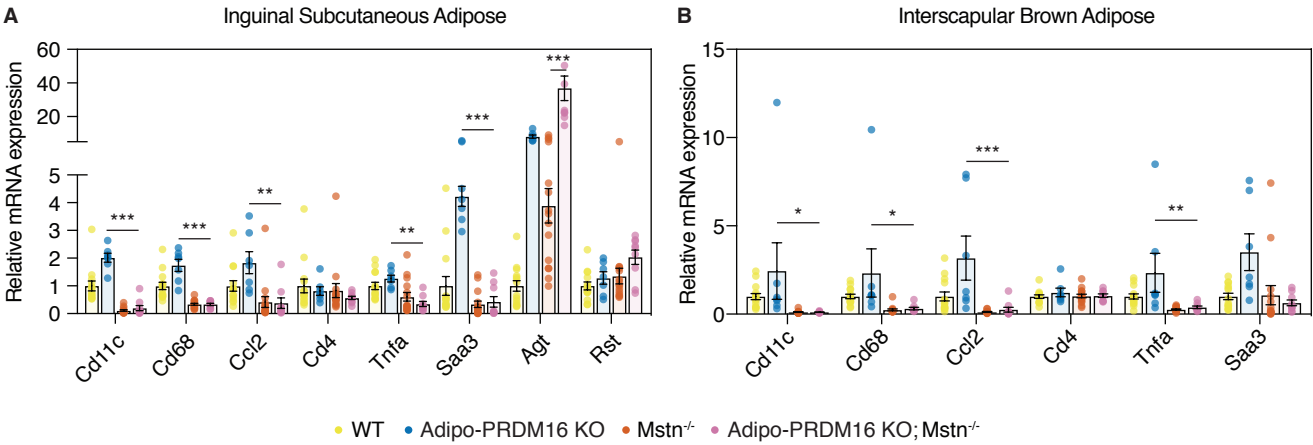

Supplement: Multimedia component 7 — Myostatin deletion protects from adipose inflammation independent of beige fat activation. Male Adipo-PRDM16 KO; Mstn-/- and control mice fed 18 weeks on HFD. (A) iWAT mRNA expression of monocyte/macrophage, T cell and pro-inflammatory markers determined by qPCR. n = 7-15 per group, ∗∗p < 0.01 ∗∗∗p < 0.001 by one-way ANOVA with Tukey post-hoc test. (B) iBAT mRNA expression of monocyte/macrophage, T cell and pro-inflammatory markers determined by qPCR. n = 7-15 per group, ∗p < 0.05 ∗∗p < 0.01 ∗∗∗p < 0.001 by one-way ANOVA with Tukey post-hoc test. [file mmc7.pdf]
