# Supplementary material for: Beige fat is dispensable for the metabolic benefits associated with myostatin deletion
Source: Mol Metab. 2020 Nov 18;43:101120. doi: 10.1016/j.molmet.2020.101120 (PMC7736974; doi:10.1016/j.molmet.2020.101120)
Supplement: Multimedia component 8 [file mmc8.docx]

**Table S1. Phenotypic characterization of male Adipo-PRDM16 KO; Mstn^-/-^ double mutant and controls on chow diet**

|  | WT  (n = 13) | Adipo-PRDM16 KO  (n = 10) | Mstn^-/-^  (n = 13) | Adipo-PRDM16 KO  Mstn^-/-^  (n = 13) | P-Value  (dKO vs Adipo-PRDM16 KO) | P-Value  (dKO vs Mstn^-/-^) |
| --- | --- | --- | --- | --- | --- | --- |
| Body Weight (g) | 25.5 ± 1.7 | 27.2 ± 1.2 | 32.0 ± 2.4 | 30.6 ± 3.4 | 0.008 | NS |
| Food Consumption (kcal/day) | 11.9 ± 0.4 | 13.2 ± 1.2 | 12.6 ± 0.5 | 12.9 ± 0.8 | NS | NS |
| Fasted Blood Glucose (mg/dL) | 153 ± 18 | 146 ± 19 | 149 ± 22 | 151 ± 15 | NS | NS |
| Body Length (cm) | 17.1 ± 0.5 | 17.2 ± 0.3 | 16.8 ± 0.4 | 16.7 ± 0.6 | 0.045 | NS |
| Adiposity (%) | 8.5 ± 1.9 | 8.2 ± 2.4 | 4.4 ± 2.1 | 4.4 ± 1.8 | 0.0003 | NS |
| Body Fat Mass (g) | 2.2 ± 0.5 | 2.2 ± 0.6 | 1.4 ± 0.7 | 1.3 ± 0.5 | 0.0026 | NS |
| Leanness (%) | 83.8 ± 2.7 | 83.4 ± 2.9 | 92.0 ± 4.5 | 90.8 ± 2.1 | <0.0001 | NS |
| Body Non-Fat Mass (g) | 21.4 ± 1.6 | 22.7 ± 1.4 | 29.4 ± 2.7 | 27.8 ± 3.2 | <0.0001 | NS |
| Brown Adipose (g) | 0.03 ± 0.01 | 0.03 ± 0.01 | 0.03 ± 0.01 | 0.04 ± 0.01 | 0.0078 | NS |
| Inguinal Adipose (g) | 0.14 ± 0.03 | 0.16 ± 0.03 | 0.11 ± 0.02 | 0.13 ± 0.02 | NS | NS |
| Epididymal Adipose (g) | 0.13 ± 0.04 | 0.13 ± 0.03 | 0.10 ± 0.03 | 0.09 ± 0.03 | 0.0029 | NS |
| Tibialis Anterior Muscle (g) | 0.05 ± 0.01 | 0.06 ± 0.01 | 0.09 ± 0.01 | 0.09 ± 0.01 | <0.0001 | NS |
| Gastrocnemius Muscle (g) | 0.14 ± 0.01 | 0.14 ± 0.02 | 0.28 ± 0.04 | 0.27 ± 0.05 | <0.0001 | NS |

Values are mean ± SD

dKO: Double knockout

**Table S2. Phenotypic characterization of female Adipo-PRDM16 KO; Mstn^-/-^ double mutant and controls on chow diet**

|  | WT  (n = 9) | Adipo-PRDM16 KO  (n = 10) | Mstn^-/-^  (n = 9) | Adipo-PRDM16 KO  Mstn^-/-^  (n = 11) | P-Value  (dKO vs Adipo-PRDM16 KO) | P-Value  (dKO vs Mstn^-/-^) |
| --- | --- | --- | --- | --- | --- | --- |
| Body Weight (g) | 22.5 ± 0.9 | 22.4 ± 1.4 | 26.3 ± 1.5 | 25.4 ± 1.4 | <0.0001 | NS |
| Food Consumption (kcal/day) | 11.0 ± 0.5 | 11.2 ± 0.9 | 11.0 ± 1.9 | 11.5 ± 0.9 | NS | NS |
| Fasting Blood Glucose (mg/dL) | 138 ± 14 | 141 ± 13 | 147 ± 21 | 146 ± 9 | NS | NS |
| Body Length (cm) | 17.4 ± 0.6 | 17.2 ± 0.5 | 16.5 ± 0.3 | 16.4 ± 0.5 | 0.0064 | NS |
| Adiposity (%) | 13.6 ± 2.3 | 11.9 ± 3.3 | 7.2 ± 1.5 | 7.0 ± 0.9 | 0.0003 | NS |
| Body Fat Mass (g) | 3.06 ± 0.57 | 2.68 ± 0.83 | 1.89 ± 0.37 | 1.79 ± 0.28 | 0.0091 | NS |
| Leanness (%) | 78.9 ± 3.4 | 80.5 ± 5.3 | 86.4 ± 1.8 | 89.0 ± 1.3 | <0.0001 | NS |
| Body Non-Fat Mass (g) | 17.73 ± 0.97 | 17.99 ± 0.94 | 22.72 ± 1.47 | 22.64 ± 1.33 | <0.0001 | NS |
| Brown Adipose (g) | 0.02 ± 0.01 | 0.02 ± 0.01 | 0.02 ± 0.01 | 0.02 ± 0.01 | NS | NS |
| Inguinal Adipose (g) | 0.16 ± 0.05 | 0.16 ± 0.06 | 0.12 ± 0.02 | 0.12 ± 0.04 | NS | NS |
| Parametrial Adipose (g) | 0.16 ± 0.05 | 0.09 ± 0.04 | 0.09 ± 0.03 | 0.07 ± 0.02 | NS | NS |
| Tibialis Anterior Muscle (g) | 0.03 ± 0.01 | 0.03 ± 0.00 | 0.06 ± 0.01 | 0.06 ± 0.01 | <0.0001 | NS |
| Gastrocnemius Muscle (g) | 0.12 ± 0.01 | 0.11 ± 0.01 | 0.19 ± 0.03 | 0.20 ± 0.03 | <0.0001 | NS |

Values are mean ± SD

dKO: Double knockout

**Table S3. Phenotypic characterization of female Adipo-PRDM16 KO; Mstn^-/-^ double mutant and controls on HFD**

|  | WT  (n = 11) | Adipo-PRDM16 KO  (n = 8) | Mstn ^-/-^  (n = 7) | Adipo-PRDM16 KO; Mstn ^-/-^  (n = 12) | P-Value  (dKO vs Adipo-PRDM16 KO) | P-Value  (dKO vs Mstn^-/-^) |
| --- | --- | --- | --- | --- | --- | --- |
| Body Weight (g) | 40.55 ± 4.56 | 47.76 ± 5.19 | 30.05 ± 1.13 | 33.31 ± 2.71 | <0.0001 | NS |
| Fasting Blood Glucose (mg/dL) | 185 ± 19 | 173 ± 17 | 146 ± 13 | 142 ± 21 | 0.0031 | NS |
| Adiposity (%) | 45.13 ± 4.27 | 49.54 ± 5.43 | 10.06 ± 5.10 | 9.96 ± 4.56 | <0.0001 | NS |
| Body Fat Mass (g) | 18.45 ± 3.80 | 23.84 ± 4.44 | 3.06 ± 1.52 | 3.41 ± 1.79 | <0.0001 | NS |
| Leanness (%) | 50.41 ± 4.69 | 45.36 ± 5.12 | 83.71 ± 3.62 | 83.90 ± 4.12 | <0.0001 | NS |
| Body Non-Fat Mass (g) | 20.30 ± 1.50 | 21.50 ± 1.75 | 25.49 ± 1.51 | 27.86 ± 1.48 | <0.0001 | 0.011 |
| Brown Adipose (g) | 0.06 ± 0.01 | 0.08 ± 0.03 | 0.02 ± 0.01 | 0.03 ± 0.01 | <0.0001 | NS |
| Inguinal Adipose (g) | 1.73 ± 0.43 | 2.29 ± 0.56 | 0.29 ± 0.09 | 0.51 ± 0.30 | <0.0001 | NS |
| Parametrial Adipose (g) | 1.59 ± 0.48 | 1.62 ± 0.30 | 0.13 ± 0.06 | 0.19 ± 0.12 | <0.0001 | NS |
| Tibialis Anterior Muscle (g) | 0.05 ± 0.01 | 0.05 ± 0.01 | 0.10 ± 0.02 | 0.11 ± 0.02 | <0.0001 | NS |
| Liver (g) | 1.53 ± 0.34 | 1.23 ± 0.19 | 0.80 ± 0.06 | 0.90 ± 0.13 | 0.0130 | NS |

Values are mean ± SD

dKO: Double knockout

**Table S4. Clinical blood chemistry of male Adipo-PRDM16 KO; Mstn^-/-^ double mutant and controls on HFD**

|  | WT  (n = 14) | Adipo-PRDM16 KO  (n = 7) | Mstn^-/-^  (n = 15) | Adipo-PRDM16 KO;  Mstn^-/-^  (n = 9) | P-Value  (dKO vs Adipo-PRDM16 KO ) | P-Value  (dKO vs Mstn^-/-^) |
| --- | --- | --- | --- | --- | --- | --- |
| GDF8 (ng/ml) | 37.1 ± 8.4 | 42.7 ± 7.6 | 0 ± 0 | 0 ± 0 | <0.0001 | NS |
| Glycemia (mg/dL) | 229 ± 30 | 219 ± 18 | 167 ± 14 | 164 ± 21 | <0.0001 | NS |
| Insulin (ng/ml) | 1.1 ± 0.9 | 1.1 ± 0.6 | 0.4 ± 0.2 | 0.4 ± 0.2 | 0.05 | NS |
| Triglycerides (mg/dl) | 47.7 ± 10.5 | 45.2 ± 9.7 | 45.4 ± 9.0 | 42.3 ± 12.4 | NS | NS |
| NEFA (mEq/dl) | 5.11 ± 1.34 | 6.60 ± 1.78 | 5.68 ± 2.16 | 5.67 ± 2.96 | NS | NS |
| Lactate (mM) | 14.14 ± 1.66 | 13.19 ± 0.97 | 13.68 ± 1.65 | 13.76 ± 1.81 | NS | NS |

Mice were fasted 5-6 hrs

Values are mean ± SD

dKO: Double knockout
